# Metabolome and Transcriptome Analysis Revealed the Basis of the Difference in Antioxidant Capacity in Different Tissues of *Citrus reticulata* ‘Ponkan’

**DOI:** 10.3390/antiox13020243

**Published:** 2024-02-18

**Authors:** Xiao Liang, Huixin Wang, Wanhua Xu, Xiaojuan Liu, Chenning Zhao, Jiebiao Chen, Dengliang Wang, Shuting Xu, Jinping Cao, Chongde Sun, Yue Wang

**Affiliations:** 1 Laboratory of Fruit Quality Biology, The State Agriculture Ministry Laboratory of Horticultural Plant Growth, Development and Quality Improvement, Zhejiang Provincial Key Laboratory of Integrative Biology of Horticultural Plants, Zhejiang University, Hangzhou 310058, China; 11816039@zju.edu.cn (X.L.); 22016155@zju.edu.cn (H.W.); 22116039@zju.edu.cn (W.X.); 11416048@zju.edu.cn (X.L.); 11616041@zju.edu.cn (C.Z.); jiebiaochen@zju.edu.cn (J.C.); 0017165@zju.edu.cn (J.C.); adesun2006@zju.edu.cn (C.S.); 2 Citrus Research Institute, Quzhou Academy of Agricultural Sciences, Quzhou 324000, China; yuewa@zju.edu.cn; 3 Hangzhou Agriculture Technology Extension Center, Hangzhou 310058, China; shutingxu@zju.edu.cn

**Keywords:** citrus, antioxidant capacity, secondary metabolites, transcriptome, WGCNA

## Abstract

Citrus is an important type of fruit, with antioxidant bioactivity. However, the variations in the antioxidant ability of different tissues in citrus and its metabolic and molecular basis remain unclear. Here, we assessed the antioxidant capacities of 12 tissues from *Citrus reticulata* ‘Ponkan’, finding that young leaves and root exhibited the strongest antioxidant capacity. Secondary metabolites accumulated differentially in parts of the citrus plant, of which flavonoids were enriched in stem, leaf, and flavedo; phenolic acids were enriched in the albedo, while coumarins were enriched in the root, potentially explaining the higher antioxidant capacities of these tissues. The spatially specific accumulation of metabolites was related to the expression levels of biosynthesis-related genes such as *chalcone synthase* (*CHS*), *chalcone isomerase* (*CHI*), *flavone synthase* (*FNS*), *O-methyltransferase* (*OMT*), *flavonoid-3′-hydroxylase* (*F3′H*), *flavonoid-6/8-hydroxylase* (*F6/8H*), *p-coumaroyl CoA 2′-hydroxylase* (*C2′H*), and *prenyltransferase* (*PT*), among others, in the phenylpropane pathway. Weighted gene co-expression network analysis (WGCNA) identified modules associated with flavonoids and coumarin content, among which we identified an OMT involved in coumarin *O*-methylation, and related transcription factors were predicted. Our study identifies key genes and metabolites influencing the antioxidant capacity of citrus, which could contribute to the enhanced understanding and utilization of bioactive citrus components.

## 1. Introduction

Oxidation is the process of producing free radicals, which often contribute to aging and the development of chronic diseases, and it can be slowed down or even inhibited by antioxidants [1]. At present, natural antioxidants from plants have attracted significant interest in human health care as well as disease prevention and have found wide applications in food, drugs, additives, cosmetics, and other fields [2,3]. Citrus, belonging to the genus *Citrus* L. of the family *Rutaceae*, is an important fruit crop cultivated extensively worldwide with significant production [4]. Citrus is enriched with natural antioxidants in addition to vitamin C in the flesh and abundant secondary metabolites distributed in other tissues, such as phenolic acids, flavonoids, coumarins, limonoids, alkaloids, carotenoids and essential oils, all of which possess notable antioxidant capacity [5]. These secondary metabolites participate in important physiological processes such as stress resistance, disease resistance and signal transduction in plants [6], and are also vital sources of dietary antioxidants.

Citrus have been used as traditional medicinal herbs in Asian countries [5], with various parts, including peels, leaves, and seeds, being used for medicinal purposes. Currently, several studies have compared the antioxidant function of different tissues of citrus, finding that the peel and seed showed more prominent antioxidant capacity than the pulp [7]. However, the antioxidant capacity of roots, stems, leaves, and other parts of the fruit have seldom been assessed. The antioxidant capacity of different tissues is intricately linked to the accumulation of bioactive components. Secondary metabolites, known for their diverse bioactive functions, demonstrate a spatially specific distribution in citrus. For example, polymethoxylated flavones (PMFs) have been widely detected in the flavedo and limonoids have been found in the segment membranes and seeds, while flavonoids, alkaloids, and coumarins have been detected in the flowers and fruits [8]. However, a detailed illustration of the secondary metabolome profile in the citrus plant is still lacking. Due to processing or pruning every year, there are many by-products of citrus, such as peels, seeds, pomace, branches, and leaves [9], all of which are believed to be rich sources of natural bioactive substance and antioxidants. Utilizing these by-products scientifically could reduce waste and further enhance the health care potential of citrus.

At present, the biosynthesis pathways of secondary metabolites in plants have been extensively studied, including flavonoids [10], coumarins [11], phenolic acids [12], terpenoids [13], and alkaloids [14]. However, knowledge of the biosynthesis pathway of secondary metabolites in citrus is still incomplete, especially the transcriptional regulation mechanism, which remains unclear. The combination of metabolome and transcriptome analysis is recognized as an effective approach to compare the metabolic diversity between samples as well as to reveal related molecular mechanisms [15]. Weighted gene co-expression network analysis (WGCNA) is an efficient tool for identifying cluster of genes with similar functions based on their correlation, thus facilitating the construction of predictive regulatory networks [16]. WGCNA combined with metabolome data is a powerful tool for identifying the genetic basis of the specific accumulation of secondary metabolites in citrus.

In this study, we evaluated and compared the antioxidant capacity of 12 tissues from *Citrus reticulata* ‘Ponkan’ (hereinafter referred to as ‘Ponkan’), a widely recognized citrus variety in China, using four chemical assays. Through metabolome analysis, we outlined a comprehensive metabolic profile of citrus plant and analyzed possible secondary metabolites that are related to antioxidant functions in different tissues. Through combined transcriptomic and metabolomic analysis as well as WGCNA, we excavated genes related to the accumulation and regulation of citrus secondary metabolite synthesis. Our research will contribute to the full utilization of citrus resources and the development of the medicinal value of citrus plants.

## 2. Materials and Methods

### 2.1. Plant Materials

**Figure 1:**
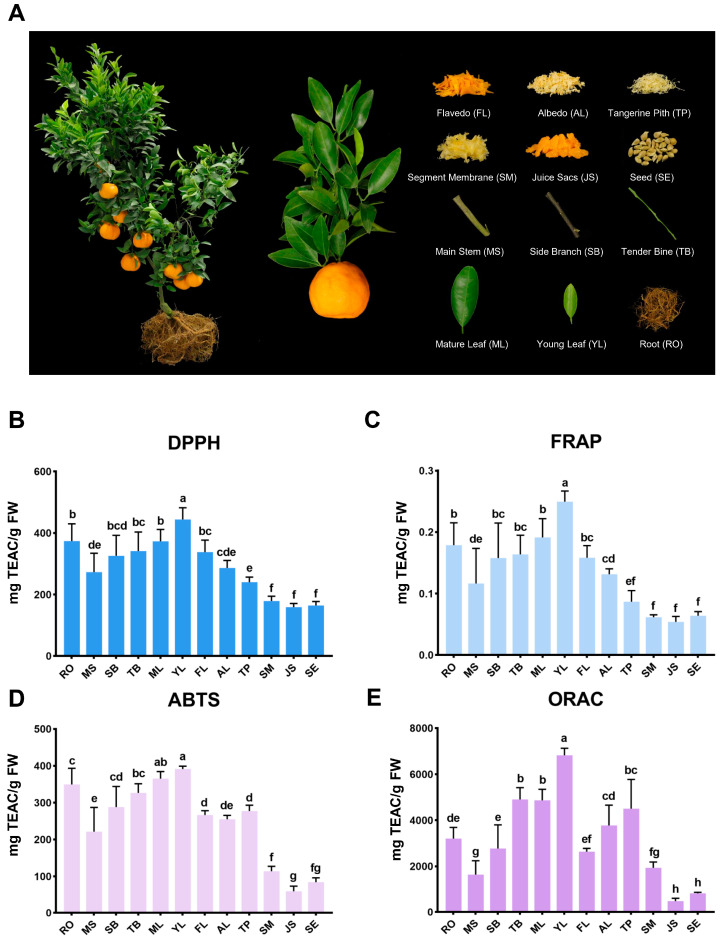
Comparison of in vitro antioxidant capacity of 12 Ponkan tissue extracts. (**A**) The whole plant and 12 different Ponkan tissues. (**B**–**E**) The antioxidant index (represented by mg TEAC/g FW) of 12 Ponkan tissues evaluated by DPPH (**B**), FRAP (**C**), ABTS (**D**), and ORAC (**E**). Different lowercase letters on the column represent statistically significant differences at the 0.05 probability level.

Three whole Ponkan plants were harvested in Quzhou, Zhejiang Province. As shown in Figure 1A, the tissues including flavedo (FL), albedo (AL), tangerine pith (TP), segment membranes (SM), juice sacs (JS), seeds (SE), main stems (MS), side branches (SB), tender bines (TB), mature leaves (ML), young leaves (YL), and roots (RO) were isolated from various parts of each plant. Three plants served as three biological replicates for each tissue sample. The samples were rapidly frozen in liquid nitrogen and stored at −80 °C, then lyophilized by vacuum freeze-dryer (Scientz-100F, Scientz, Ningbo, China) and ground using a mixer mill (MM 400, Retsch, Haan, Germany) with a zirconia bead (1.5 min, 30 Hz). A 100 mg quantity of lyophilized powder was added to 1.2 mL 70% methanol solution, and then vortexed for 30 s per 30 min for 6 times in total, then finally placed at 4 °C overnight. The extracts were collected by high-speed centrifugation (12,000 rpm, 10 min) and filtrated through filtration membrane before UPLC-MS/MS analysis.

### 2.2. In Vitro Chemical Antioxidant Capacity Evaluation

Ponkan extract preparation: For each tissue, 0.2 g of fresh sample powder was accurately weighed and combined with 2 mL methanol. Ultrasonication-assisted extraction was performed for 30 min followed by centrifugation (8000 rpm,10 min), repeated twice, and the supernatants were combined. Four chemical antioxidant capacity evaluation assays were employed to evaluate the in vitro antioxidant capacities of Ponkan extracts, including 2,2-diphenyl-1-picrylhydrazyl (DPPH) free radical scavenging assay, ferric ion-reducing antioxidant power (FRAP) assay, 2,2′-azino-bis(3-ethylbenzothiazoline-6-sulfonic acid) (ABTS) radical scavenging assay and oxygen radical absorbance capacity (ORAC) assay, and the detailed operation method was referred to Wang et al. [17]. The test of each sample repeated three times and all the results were expressed as mg Trolox equivalent antioxidant capacity (TEAC)/g FW (fresh weight).

### 2.3. Metabolite Profiling Using UPLC-MS/MS

The metabolome analysis was performed by Wuhan Metware Biotechnology Co., Ltd., Wuhan, China, following the methods created by Chen et al. [18]. The sample extracts were analyzed using a UPLC-ESI-MS/MS system (UPLC, SHIMADZU Nexera X2, SHIMADZU, Kyoto, Japan; MS, Applied Biosystems 4500 Q TRAP, AB SCIEX, Framingham, MA, USA) with the following specifications: UPLC column, Agilent SB-C18 (1.8 µm, 2.1 mm × 100 mm); solvent A, water (0.1% formic acid); solvent B, acetonitrile (0.1% formic acid); gradient program, 0/95, 9/5, 10/5, 11.1/95 and 14/95. (min/A%); flow rate, 0.35 mL/min; injection volume, 4 μL. LIT and triple quadrupole (QQQ) scans were acquired on a triple quadrupole–linear ion trap mass spectrometer (Q TRAP), AB4500 Q TRAP UPLC/MS/MS System, equipped with an ESI Turbo Ion-Spray interface, operating in positive and negative ion mode and controlled by Analyst 1.6.3 software (ABSCIEX, Framingham, MA, USA). The reference database for metabolite identification was Metware Database (MWDB), created by Wuhan Metware Biotechnology Co., Ltd. The identification of metabolites was synthetically based on the precise mass of metabolites, the information of MS2 fragments, the isotopic distribution of MS2 fragments, and the retention time (RT). Through the intelligent matching method independently developed by Metware, the MS2 information and RT of metabolites in the sample were intelligently matched with the database one by one. The MS and MS2 tolerances were set to 20 ppm, and the RT tolerances were set to 0.2 min. The differentially accumulated metabolites (DAMs) were identified based on variable importance in projection (VIP) ≥ 1 and fold-change (FC) ≥ 2 or ≤0.5.

### 2.4. RNA-Seq Analysis

A total of 36 high-quality RNA samples were used to create sequencing libraries using the NEBNext UltraTM RNA Library PrepKit for Illumina (NEB, Beverly, MA, USA). Then the library sequencing was performed on an Illumina NovaSeq 6000 platform. After data filtering by fastp v0.19.3, the clean reads were aligned to the Citrus clementina v1.0 genome using HISAT v2.1.0. Gene expression level was evaluated by fragments per kilobase of transcript per million fragments mapped (FPKM), which was calculated using feature Counts v1.6.2. DESeq2 v1.22.1 was used to identify differentially expressed genes (DEGs) between different groups, and the Benjamin and Hochberg method was used to correct *p*-value. Genes with fold-change (FC) ≥ 2 or ≤0.5 and FDR < 0.05 were assigned as DEGs.

### 2.5. RT-qPCRAnalysis

Total RNA samples were extracted as described above, and cDNA was synthesized using a PrimeScript RT reagent kit with a gDNA remover (TaKaRa, Dalian, China). The qRT-PCR was performed by CFX96™ Real-Time System (C1000™ thermal cycler, Bio-Rad, Hercules, CA, USA). Primers were designed in NCBI Primer-BLAST (https://www.ncbi.nlm.nih.gov/tools/primer-blast/, accessed on 1 January 2024). Citrus β-actin was selected as an internal standard, and for each tissue, three replicates were used to calculated the relative expression level (represented by ΔCt).

### 2.6. Weighted Gene Co-Expression Network Analysis

WGCNA v1.69 was used for weighted gene co-expression network analysis. The FPKM expression file was filtered using the varfilter function of the genefilter package in R before WGCNA. The soft threshold was set at 19, and a similar module merge threshold was set at 0.25. Genes with a high co-expression connection within the module were filtered, and Cytoscape was used to construct a co-expression network illustration.

### 2.7. Recombinant Protein Construction and Enzyme Assay

The coding sequences (CDSs) of OMT-2 from Ponkan were cloned into the pET32a expression vector with a histidine tag, followed by the recombinant vectors transferred into BL21(DE3) cells (Promega, Madison, WI, USA). The bacteria were cultured in LB medium containing 0.1g·L^−1^ ampicillin until the OD600 reached 0.8. The induced expression of recombinant proteins was carried out by the addition of isopropyl-β-D-thiogalactopyranoside (IPTG) for 24 h at 16 °C, then the bacteria cells were collected by centrifugation (4000 rpm, 4 °C, 10 min) and resuspended by 1× PBS buffer. After disruption by a sonicator, the cell debris was discarded, while the supernatant was collected by centrifugation (5000 rpm, 4 °C, 15 min). Recombinant proteins were purified using a HisTALON™ Gravity Columns Purification Kit (Takara, Dalian, China) and finally eluted in Tris-HCl buffer (50 mM, pH 8.0) containing 10% glycerol and 2 mM DTT. SDS-PAGE was used to confirm the target recombinant proteins. The in vitro enzyme assays were performed in a total volume of 200 μL consisting of 1 mM SAM, 200 μM substrates, and 25 μM of purified recombinant proteins in Tris-HCl buffer (50 mM, pH 8.0) at 37 °C for 3h, and finally stopped by adding an equal volume of methanol. After concentration by a rotary evaporator (Eppendorf, Hamburg, Germany), the precipitate was redissolved with 100 μL methanol, and the supernatant collected by high-speed centrifugation (12,000 rpm, 15 min) was ready for LC-MS detection.

HPLC conditions were as follows: detection system, Waters e2625 system equipped with a DAD detector coupled with an ODS C18 column (SunFire 5 μm, 4.6 × 250 mm, Waters, Milford, MA, USA); flow rate, 1 mL/min; solvent A, acetonitrile; solvent B, water (0.1% formic acid); gradient program, 0/20, 3/20, 5/30, 10/30, 15/40, 20/60, 23/80, 25/100, 27/20, 30/20. (min/A%); injection volume, 10 μL; UV detection wavelength, 350 nm. For MS analysis, an AB Triple TOF 5600 plus System (AB SCIEX, Framingham, MA, USA) was used and MS spectra were obtained in negative ion mode or positive ion mode (ESI). The products were identified by the retention time compared with the standards as well as the ion fragments.

### 2.8. Statistical Analysis

Statistical significance analysis for results 3.1 was conducted using one-way analysis of variance (ANOVA) using SPSS version 23.0 (IBM, Armonk, NY, USA). Principal component analysis (PCA), heatmaps, venn diagrams and volcano pots were conducted using online tools of Metware Cloud (https://cloud.metware.cn, accessed on 1 January 2024).

## 3. Results

### 3.1. Antioxidant Capacity Evaluation of 12 Ponkan Tissues

A total of 12 Ponkan tissues were separated and collected, comprising flavedo (FL), albedo (AL), tangerine pith (TP), segment membranes (SM), juice sacs (JS), seeds (SE), main stems (MS), side branches (SB), tender bines (TB), mature leaves (ML), young leaves (YL), and roots (RO) (Figure 1A). Four chemical assays were applied to evaluate the antioxidant capacities of the extracts from 12 Ponkan tissues. As shown in Figure 1B–E, in the DPPH assay, the antioxidant capacities of the tissues varied significantly, between 159 and 443 mg TEAC/g FW. YL showed the highest DPPH value while JS, SE, and SM had the lowest values, with no significant difference. In the FRAP assay, the antioxidant activity of Ponkan tissues ranged significantly, from 0.05 to 0.24 mg TEAC/g FW. Similar to the DPPH assay, the highest value was observed in YL, while the JS, SE, and SM were the lowest. ABTS values ranged significantly, from 59.58 to 365.35 mg TEAC/g FW. YL showed the highest ABTS values, while JS and SE showed the lowest values. In the ORAC assay, the ORAC values varied significantly, from 488.86 to 6821.72 mg TEAC/g FW. YL had the highest ORAC values, while JS and SE had the lowest values. In conclusion, based on the results from the four antioxidant assays, YL had the strongest antioxidant capacity, followed by RO; and SB, TB, MS, and FL also exhibited excellent antioxidant capacity. In contrast, JS and SE demonstrated the weakest antioxidant capacities.

### 3.2. The Metabolic Basis of Different Antioxidant Capacity in Ponkan Tissues

**Figure 2:**
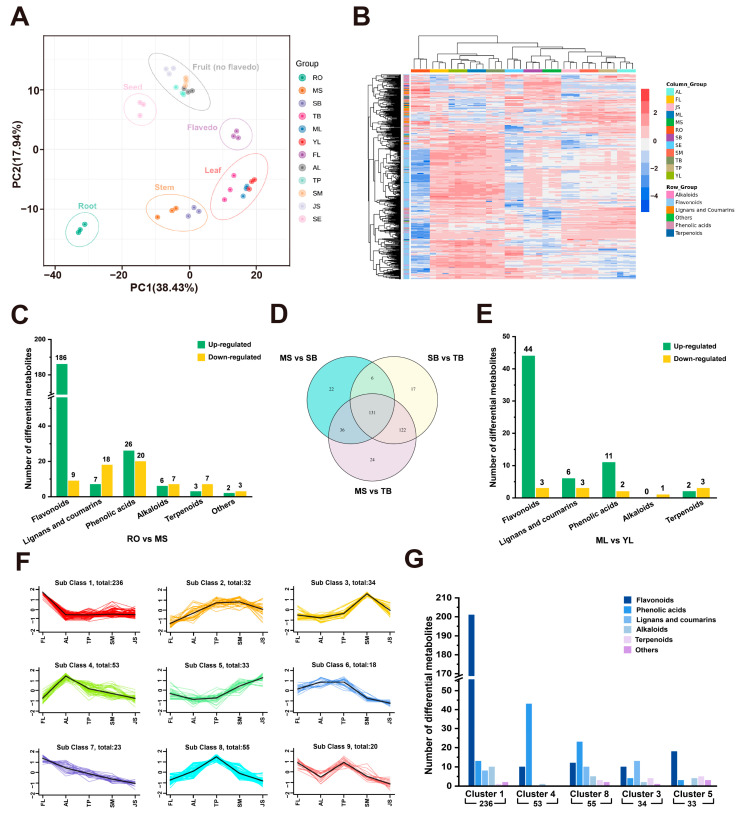
Metabolomic profiling of Ponkan. (**A**) PCA based on secondary metabolome from 12 Ponkan tissues. (**B**) Clustering heatmap of the secondary metabolites detected in 12 Ponkan tissues. (**C**) Statistics of different classes of up-regulated and down-regulated metabolites in the comparison of RO vs. MS. (**D**) Venn diagram of DAMs shared among the comparison of MS vs. SB, SB vs. TB, and MS vs. TB. (**E**) Statistics of different classes of up-regulated and down-regulated metabolites in the comparison of ML vs. YL. (**F**) K-means clustering grouped the metabolites from fruit tissues of Ponkan into 9 clusters. (**G**) Classification and count of DAMs in cluster 1, 4, 8, 3 and 5.

A total of 512 secondary metabolites, including 288 flavonoids, 108 phenolic acids, 51 lignans and coumarins, 32 alkaloids, 20 terpenoids, and 13 other compounds were detected in 12 Ponkan tissues, of which flavonoids accounted for the largest proportion (Figure S1, Table S1). Among all tissues, TB contained the largest amounts of secondary metabolites, followed by FL, YL, and ML, while RO contained the fewest secondary metabolites (Figure S2). The principal component analysis (PCA) of the metabolites showed that 12 tissues could be clustered into 6 groups according to the classification of root, stem, leaf, fruit, and seed (Figure 2A). FL was distinct from the other fruit tissues, indicating its distinctive metabolic characteristics. Unlike the other two stem tissues, TB wasclustered in the leaf group, suggesting that their metabolic profile was more similar to ML and YL. A cluster heatmap analysis showed that the three biological replicates of each group clustered together (Figure 2B). Among them, compared with other tissues, most of the flavonoids were enriched in FL, YL, ML, and TB, while most of the lignans and coumarins were enriched in RO.

To uncover the potential relationship between antioxidant capacity and secondary metabolites in different tissues, we explored the detail metabolic profile in four main parts: root, stem, leaf, and fruit. RO and MS are representative tissues of the underground and above-ground parts of citrus plants, respectively, and RO hassignificantly higher antioxidant capacity than MS. A total of 294 differentially accumulated metabolites (DAMs) in total were identified in the comparison of RO vs. MS, including 230 up-regulated DAMs and 64 down-regulated DAMs (Figure S3A), of which the up-regulated DAMs mainly included flavonoids (Figure 2C). The amount of lignans and coumarins down-regulated in RO vs. MS was much higher than that of up-regulated ones, indicating that RO accumulated more lignans and coumarins than MS (Figure 2C); thus, we speculated that lignans and coumarins contributed significantly to the antioxidant capacity of RO. Within stem tissues, 195, 276, and 313 DAMs were identified in the comparisons of MS vs. SB, SB vs. TB, and MS vs. TB, respectively (Figure S3B–D), in which flavonoids accounted for the most in up-regulated DAMs. The three comparison groups contained 131 common DAMS (Figure 2D), 111 of which were flavonoids that all increased gradually in the structure of MS—SB—TB, in that order (Figures S4 and S5). This indicates that flavonoids accumulate gradually from mature tissues to tender tissues in the stem, which conforms to the trend of antioxidant capacities (TB > SB > MS), demonstrating a correlation between flavonoids and antioxidant capacity. The same situation happened in leaf tissue, where YL up-regulated more metabolites, especially flavonoids, than ML and also showed stronger antioxidant capacity (Figure 2E and Figure S3E).

We grouped the fruit tissues (excluding SE) together and performed k-means analysis based on the metabolites of five tissues: FL, AL, TP, SM, and JS. All the metabolites were divided into 9 distinct clusters, of which Cluster 1, Cluster 4, Cluster 8, Cluster 3, and Cluster 5 contained metabolites that were specifically enriched in FL, AL, TP, SM, and JS, respectively (Figure 2F, Table S2). There were 236 secondary metabolites specifically enriched in FL, and the proportion of flavonoids was overwhelmingly dominant. A total of 53 metabolites were enriched specifically in AL, most of which were phenolic acids. Therefore, we believe that the metabolites that contribute to the antioxidant capacity of FL and AL may come from different classes, with flavonoids for FL and phenolic acids for AL. For TP, phenolic acids, flavonoids, and coumarins may be grouped together as contributors to antioxidant capacity. Compared to the three tissues, SM and JS—the main edible parts—were enriched in fewer secondary metabolites (34 and 33, respectively) and therefore had lower antioxidant levels. We hypothesize that there may be more accumulation of nutrients in these two tissues, while the peel tissues (FL + AL + TP), which are responsible for protection and defense, improve antioxidant capacity by accumulating diverse secondary metabolites.

### 3.3. Transcriptomic Profiling of Ponkan

**Figure 3:**
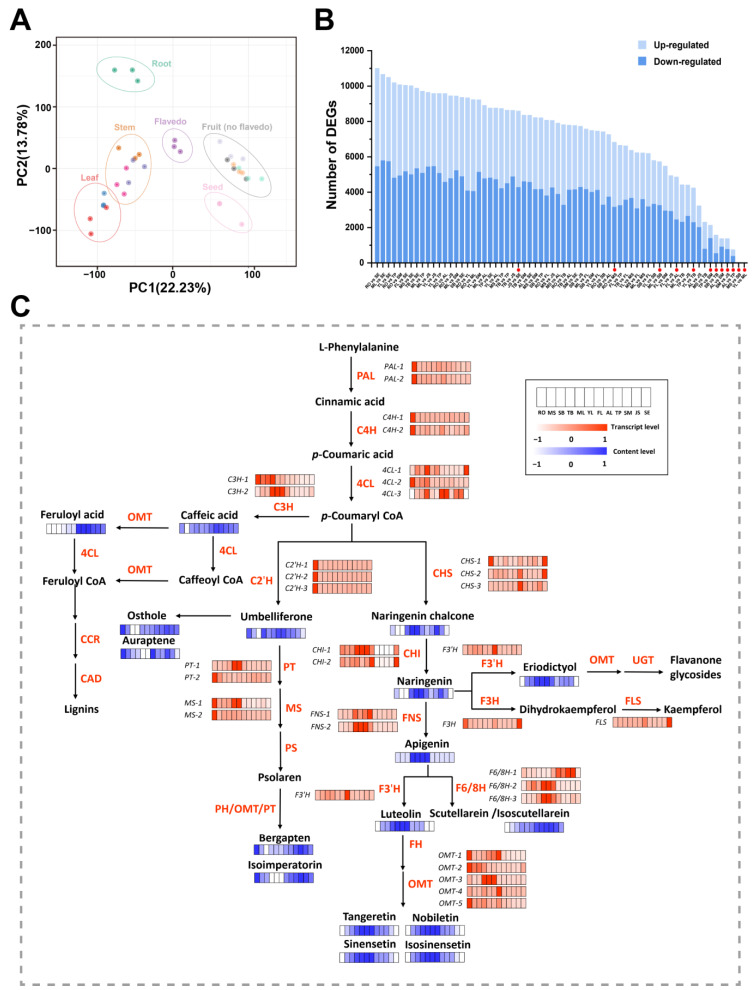
Detection and analysis of transcriptome of 12 Ponkan tissues. (**A**) PCA based on transcriptome from 12 Ponkan tissues. (**B**) Statistics of DEGs in the comparisons of all the 12 Ponkan tissues. The comparison group of adjacent tissues are marked with red dots. (**C**) Heatmap of key gene expression in flavonoid and coumarin synthesis pathways of 12 Ponkan tissues according to FPKM values. Key enzymes: PAL, phenylalanine ammonia-lyase; C4H, cinnamate 4-hydroxylase; 4CL, 4-coumarate:CoA ligase; CHS, chalcone synthase; CHI, chalcone isomerase; FNS, flavone synthase; F3H, flavanone-3-hydroxylase; F3′H, flavonoid-3′-hydroxylase; F6/8H, flavonoid-6/8-hydroxylase; OMT, *O*-methyltransferase; FLS, flavonol synthase; UGT, UDP-glycosyltransferase; C3H, coumarate 3-hydroxylase; C2′H, *p*-coumaroyl CoA 2′-hydroxylase; PT, prenyltransferase; MS, marmesin synthase; PS, psoralen synthase; PH, psoralen hydroxylase; CCR, cinnamoyl-CoA reductase; CAD, cinnamyl alcohol dehydrogenase.

To uncover the molecular basis underlying the metabolic diversity among different Ponkan tissues, transcriptome sequencing of the same samples was performed. A total of 245.08 Gb of clean data were generated from all the 36 samples, with the clean data of each sample reaching 5 Gb and the percentage of Q30 values reaching 90% or above. The PCA of the samples based on the transcriptome data showed that 12 tissues could be clustered into 6 groups according to the classification of tissues, similar to the cluster of the metabolic PCA, except that the TB samples were clustered in the stem group (Figure 3A). The results of PCA suggested that the gene expression of different Ponkan tissues showed its own characteristics and could explain the metabolic differences to a certain extent. A total of 470,612 DEGs were finally identified by the pair-wise comparisons of all the groups, shown in totality in Figure 3B. We found that there were fewer DEGs between adjacent tissues (marked with red dots in Figure 3B), while more DEGs were produced in tissues that were far away from each other.

### 3.4. Combined Transcriptomic and Metabolomic Analysis of Secondary Metabolites’ Accumulation in Citrus

Based on the results of metabolomic analysis, we suggest that the metabolites of the phenylpropane pathway play an important role in the antioxidant capacity of citrus tissues. Consequently, we selected key genes and metabolites in this pathway for further analysis (Figure 3C). The genes *PAL*s, *C4H*s, *4CL-2*, and *C3H-1* had the highest expression level in RO, which may be related to downstream accumulation of coumarins and lignans in roots. CHS and CHI are the first two key enzymes of the flavonoid biosynthesis pathway, and *CHS-2*, *CHS-3* and *CHI-2* had high expression levels in FL, while *CHI-1* was also highly expressed in FL, ML, YL, and TB. Among the downstream candidate genes, *FNS*s, *F6/8H-2*, *F6/8H-3*, and *OMT-3* were highly expressed in FL, ML, YL, and TB, while *F3′H*, *OMT-1*, and *OMT-4* were highly expressed specifically in FL. The content of flavonoid products in this pathway, such as naringenin chalcone, naringenin, eriodictyol, apigenin, luteolin, and four polymethoxylated flavonoids, were consistent with the expression level of the above genes. The high expression of the corresponding genes in YL, ML, TB, and FL explains the source of their abundant flavonoids and high antioxidant levels. Isoscutellarein accumulated in fruit tissues, and this accords with the expression characteristics of *F6/8H-1*.

The enzyme C2′H is the first step in coumarin synthesis for catalyzing its basic backbone, umbelliferone, which could further result in furocoumarin under the catalysis of a series of enzymes such as PT, MS, PS, PH, and OMT. The genes related to coumarin synthesis (including *C2′H*s, *PT-2*, and *MS*) had a high expression level in RO, and the content of typical coumarins such as umbelliferone, osthole, auraptene, bergapten, and isoimperatorin is high in RO, which endows RO with a strong antioxidant capacity compared to other tissues. Notably, three OMTs (*OMT-1*, *OMT-2*, and *OMT-5*), which have been proved to be flavonoid-related [19,20], were also found to be specifically overexpressed in ROs. Therefore, we speculated that these OMTs may not only participate in the methoxylation modification of flavonoids but also in that of coumarins.

To further confirm tissue-specific expression of key genes in the phenylpropane pathway, we performed RT-qPCR on parts of genes in six representative tissues (RO, SB, YL, FL, JS, and SE) and compared their relative expression with transcriptome levels (Figure S6). The results showed that the expression patterns of these genes were consistent with those found by transcriptome analysis, which proved that our data were accurate and reliable. The differential expression of genes between tissues generated the differentially accumulation of secondary metabolites and the set a molecular basis for differences in antioxidant levels in citrus.

### 3.5. WGCNA and Construction of Transcription Regulation Network of Flavonoids and Coumarins

**Figure 4:**
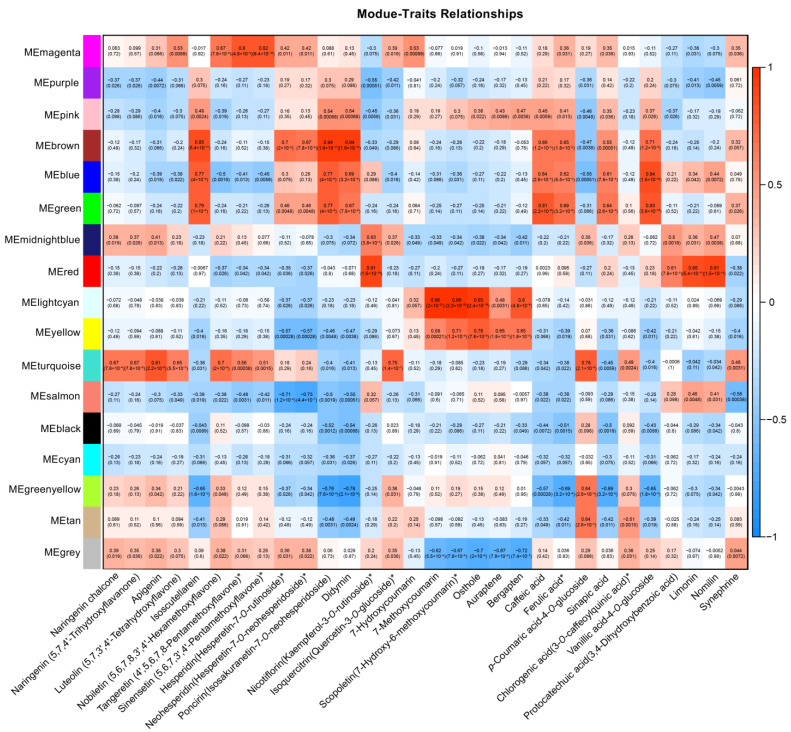
The module–trait relationships (relationships between the content of 30 selected metabolites and the module characteristic genes). In each block, the upper values denote the correlation coefficients while the lower values denote the *p*-values. The mark of “*” behind the metabolites represent probable isomers.

A total of 18,712 high-level expressed genes were selected for a weighted gene co-expression network analysis (WGCNA), leading to the identification of 17 modules based on their similar expression patterns (Figure S7A). The module–sample correlation heatmap shows that genes highly expressed in leaf tissues (ML and YL) are gathered in the turquoise module, while the genes highly expressed in FL are mainly distributed in the magenta module (Figure S7B). The module–trait correlation heatmap indicates that there are 7 and 4 flavonoid compounds highly correlated with the turquoise and magenta modules (R > 0.5 and *p*-value < 0.01), respectively. The genes highly expressed in fruit tissues (AL, TP, SM, and JS) are mainly located in the brown, blue, and green modules, and each module shows a high correlation with 3 flavonoid compounds (for the brown module, it is 5) and 4 phenolic acid compounds. The light cyan and yellow modules, which are mainly gathered the genes specifically highly expressed in RO, are correlated with 4 and 5 coumarin compounds, respectively. The red module mainly contains the genes specifically highly expressed in SE, and it was correlated with kaempferol-3-*O*-rutinoside, 3,4-dihydroxybenzoic acid, limonin, and nomilin (Figure 4). Overall, the genes gathered in different modules showed unique expression patterns corresponding to different tissues, and showed high relevance with the accumulation of specific metabolites.

**Figure 5:**
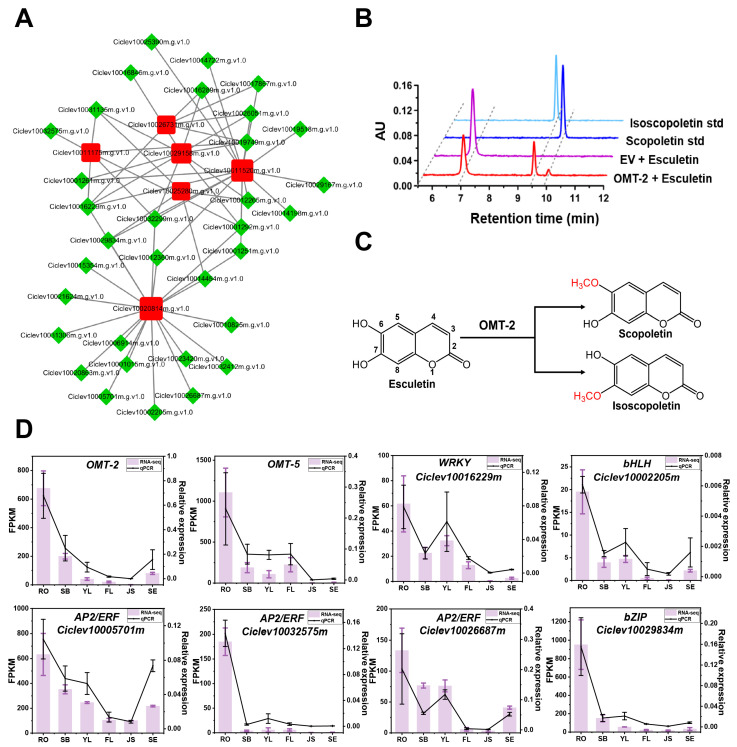
Identification of genes related to coumarin synthesis in Ponkan. (**A**) Co-expression network related to coumarins. Red nodes represent the structural genes; green diamonds represent the annotated transcription factors. (**B**) HPLC chromatograms of the in vitro reactions of OMT-2 with esculetin as substrate. (**C**) Schematic diagram of the catalytic positions of OMT-2 for esculetin. (**D**) qPCR results for the key OMTs and related TFs.

We selected the turquoise and yellow modules to construct transcription regulatory networks for flavonoids and coumarins (Figure 5A and Figure S8). We screened a series of key transcription factors (TFs) based on high connectivity (|correlation| > 0.8, *p*-value < 0.05) with hub structure genes. In the turquoise module, we identified 45 highly expressed TFs according to the network, including 2 *ERF*s, 4 *bHLH*s, 3 *NAC*s, 5 *WRKY*s, and 1 *MYB*. In the yellow module, a total of 33 TFs were predicted, containing 4 ERFs, 6 *bHLH*s, 1 *bZIP*, 2 *WRKY*s, and 2 *MYB*s. We noticed that *OMT-2* (Ciclev100201814m) and *OMT-5*(Ciclev10029158m) stayed hub genes in the coumarin-related network, and *OMT-5* has been proven to have the function of performing methylation on coumarin substrate [20], so we hypothesize that *OMT-2* possesses a similar function. We cloned the coding region of *OMT-2* and then constructed overexpression vectors. After being expressed in *E. coli*, the recombinant proteins were purified and reacted with esculetin substrate. HPLC detection and MS/MS data showed that *OMT-2* could catalyze the methoxylation of esculetin at 6- or 7-position, producing corresponding products, scopoletin and isoscopoletin (Figure 5B,C and Figure S9). The RT-qPCR analysis of *OMT-2*, *OMT-5*, and related TFs in this network confirmed their common high-expression pattern in RO (Figure 5D). These TFs may promote the accumulation of coumarin in roots by participating in the regulation of methoxylation processes.

## 4. Discussion

Over the past few decades, interest in identifying natural molecules for use as food supplements has grown, with these plant-derived bioactive substances playing a pivotal role in health care and disease prevention [2]. Citrus, a popular type of fruit, boasts numerous tissues demonstrating antioxidant potential, making it an ideal source of natural antioxidants. In this study, all the four chemical assays have been widely applied to evaluate the antioxidant capacities in natural product [21]. Comparative studies of citrus tissue antioxidant capacities have primarily focused on peel and pulp, with the peel generally exhibiting higher levels [22,23,24]. However, our study confirms that leaf tissues, particularly YL, possess the strongest antioxidant activity. Compared to fruits, citrus leaves offer advantages like broader availability and fewer seasonal limitations, indicating significant potential for development. The antioxidant activity of RO was medium to high, which we believe to be closely related to coumarin accumulation. Coumarins, as important phenolic substances, are also known for their outstanding antioxidant capacity [25]. A gradual increase in antioxidant activity was observed from MS to SB to TB, and from ML to YL, potentially due to the increased accumulation of flavonoids in younger tissues. The antioxidant capacity of the edible parts of citrus plants is relatively weak, but there exist differences between different parts. Abeysinghe et al. found that the antioxidant capacity in segment membranes is higher than that of juice sacs and segments due to the high content of total phenolics [26], aligning with our findings. In addition, we found that TP is the most antioxidant active part of the edible part, which also explains why it can be used as a traditional medicine. Contrary to previous studies, the seed did not exhibit significant antioxidant capacity in our analysis, despite being reported as rich in tannins and phenols [7]. This discrepancy may be influenced by factors such as the choice of cultivar and the stage of development.

The biological activities of citrus depend on its abundant metabolites, especially secondary metabolites [5]. Currently, both targeted and non-targeted metabolomic techniques are extensively used to detect secondary metabolites in different plant varieties [27], tissues [28], and development stages [29], and also have certain applications in citrus research [8,30]. In this study, 512 secondary metabolites from 12 Ponkan tissues were identified by widely targeted metabolomics, and the categories of metabolites detected were consistent with previous studies. The number of secondary metabolites detected in this study is much higher than previous studies, which greatly enriched the citrus secondary metabolite pool. In addition, PCA and heatmap analysis showed that the secondary metabolite profile of different tissues of citrus showed its own characteristics, which has a guiding role for the development and utilization of the medicinal value of different tissues.

Flavonoids, characterized by their high abundance and multiple biological activities, are among the most important secondary metabolites in citrus [31]. The main site for flavonoid synthesis and accumulation are oil glands, which are mainly distributed in the stems, leaves, flowers, and flavedo of fruits [32]. Therefore, leaves and stems are important sources of flavonoids. A variety of flavonoids have been identified from the leaves and stems of different citrus species [33,34]. Our research shows that leaves and tender stems are flavonoid-rich tissues, with the content YL > ML TB > SB > MS, which conforms to the conclusion that a large number of flavonoids accumulate in young and fast-growing tissues [31,35]. Flavonoid synthesis is mainly carried out in the early stages of organ and tissue development. Therefore, the utilization of citrus tender tissue appears to be more important. Coumarin is a secondary metabolite that plays an important part in the communication between plant and microbiome [36,37]. Our research shows that compared with MS, coumarins accumulate more in RO, which contributes to the improvement of rhizosphere microbial community and plant resistance. It should be emphasized that in order to increase yield and resistance, most currently planted citrus cultivars are grafted. Therefore, the root tissue used in this study is from the rootstock of trifoliate orange (*Poncirus*), so the metabolite profile of root tissue does not fully represent the characteristics of *Citrus reticulata*. In future studies, we aim to conduct further exploration using seed-derived seedlings.

At present, the research on the secondary metabolites of citrus fruits is mainly focused on flavonoids. The citrus flavedo constitutes an important source of flavonoids, comprising specific polymethoxylated flavones (PMFs) such as nobiletin, tangeretin, and sinensetin [38]. Our study shows that the number of flavonoids enriched in FL is much higher than that in other fruit tissues, which agrees with previous research [39]. In addition, the accumulation of alkaloids in the FL was also higher than that in other fruit tissues. As the FL is the tissue directly exposed to the environment of citrus fruit, the accumulation of flavonoids and alkaloids in FL may improve the fruit’s resistance to biological and abiotic stresses [40,41]. Phenolic acid is also rich in citrus peel. Previous studies have shown that in some citrus varieties, the content of phenolic acid in flavedo is much higher than that in albedo [42], but our research indicated that AL is the tissue with the richest phenolic acid accumulation. We believe that it may be caused by varietal differences. It has been reported that limonoid accumulates in segment membrane [39], but there are few studies on the secondary metabolites in tangerine pith. We found that coumarins also accumulate in TP in addition to limonoids. We believe that the detailed analysis of metabolome profile in citrus fruit will provide a valuable reference for our further utilization of specific tissues.

Combined transcriptomic and metabolomic analysis explains the specific metabolite distribution among different Ponkan tissues. PAL, 4CL, and C4H are key enzymes and rate-limiting enzymes in the phenylpropane biosynthesis pathway, closely related to the synthesis of lignins. In Arabidopsis, two *PAL*s are highly expressed in the roots, inflorescences, and stems [43], and *C4H* is also highly expressed in tissues with a high lignin content [44]; two citrus genes, *Cit4CL2* and *Cit4CL3*, are highly expressed in young stems and roots, respectively [45]. Our research showed these three genes were expressed highly in the root, which may lead to the accumulation of lignin in it.

The key enzymes of the flavonoid synthesis pathway have been extensively studied. Zhao et al. compared the relative expression levelof *CHS*s and *CHI*s in four tissues from *Citrus reticulata* ‘Ougan’, finding that they were expressed highly in young leaves and flowers, related to flavonoid content [46]. Our research shows that *CHS-2*, *CHS-3*, and *CHI-2* have the highest expression level in the FL, while *CHI-1* had the highest expression level in ML, YL, and TB, also closely related to flavonoid content. The two *FNS*s, one *F3′H*, and two *F6/8H*s selected in our study presented specific high expression in flavonoid-rich tissues, indicating their possible catalytic functions, and some of these have been reported and confirmed [47,48]. Currently, there is limited research on the synthesis of coumarins in citrus. In *Ruta graveolens*, RgC2′H has been proved to have the function of catalyzing the backbone of coumarin [11]. MS have only been reported on in fig trees [49]. For citrus, only the catalytic activity of CitPTs [50,51] and CitX5H [52] have been reported. In this research, the coumarin synthesis-related genes highly expressed in RO, such as *C2′H*s, *PT-2* and *MSs*, can be considered as candidate genes for the coumarin synthesis pathway, which may enrich the study of coumarin synthesis in citrus. OMTs have various catalytic substrates and sites, so the diversity of their expression patterns among tissues may be related to their different catalyzing functions. For example, *OMT-2* has been confirmed to have the function of catalyzing flavonoid substrates [19], conforming to its high gene expression level in FL, leaves, and stems. Meanwhile, it also had a high expression level in RO. Our in vitro enzyme reactions prove that *OMT-2* does participate in the methoxylation of esculetin, confirming its important role in the synthesis of coumarin or lignin pathway. The results show a good correlation between our transcriptome and metabolome data, as differences in metabolites between different tissues can be reasonably explained by the gene expression levels.

Numerous studies have been conducted on transcription factors related to flavonoid synthesis, primarily focusing on flavonols and anthocyanins [46]. Xu et al. found that the synthesis of anthocyanins and procyanidins in Arabidopsis is regulated by the MWB complex, composed of R2R3-MYB, basic helix loop helix (bHLH) and WD repeat proteins (WDR) [53]. Zhao et al. found that CitRAV1 forms a transcription complex with CitERF33, which enhances the transcriptional activation of *CitCHIL1* promoter and increases the content of flavonoids in citrus [46]. However, we still lack studies on the transcriptional regulation of coumarin synthesis. In this study, we constructed a co-expression network of genes highly related to flavonoid and coumarin content and excavated transcription factors strongly related to several structural genes. At present, there have been many studies on the transcriptional regulation of lignin synthesis, involving *PAL*, *C4H*, *4CL* and other genes of the phenylpropane synthesis pathway [54,55], which are also reflected in our co-expression network. For *OMT-2* and *OMT-5* that specifically expressed in roots, we screened transcription factors that may participate in methoxylation regulation according to co-expression patterns, including AR2/ERF, bZIP, bHLH, etc., which will provide new ideas for transcriptional regulation research regarding coumarin.

## 5. Conclusions

In this study, we compared the antioxidant capacities in 12 tissues of *Citrus reticulata* ‘Ponkan’. Through metabolomics analysis, the distribution characteristics of secondary metabolites in different tissue parts were analyzed, revealing that flavonoids and coumarins might be closely related to antioxidant capacity. Via transcriptomic analysis, structural genes that may lead to the tissue-specific accumulation of flavonoids and coumarins were discovered, and the transcriptional regulatory networks of flavonoids and coumarins were discovered by WGCNA. The function of OMT-2 and the co-expression trend of its related transcription factors were confirmed by in vitro enzyme activity and RT-qPCR. This study provides a basis for the enrichment and utilization of natural antioxidants in citrus as well as the study on phenylpropane pathways in plants, which will contribute to the development of citrus resources.

## Data Availability

The raw data of transcriptome involved in this study are available in the NCBI BioProject repository. BioProject ID: PRJNA954191. Other data supporting this study are included in supplementary figures and tables.

## Supplementary Materials

The following supporting information can be downloaded at: https://www.mdpi.com/article/10.3390/antiox13020243/s1, Figure S1: Classification and percentage of the identified secondary metabolites from 12 Ponkan tissues. Figure S2: Comparison of the number of secondary metabolites detected in 12 Ponkan tissues. Figure S3: Volcano pots of the DAMs from the comparisons of RO vs. MS (A), MS vs. SB (B), SB vs. TB (C), MS vs. TB (D) and ML vs. YL (E). Figure S4: The classification of 131 common metabolites that obtained by pairwise comparison of MS, SB and TB. Figure S5: K-means clustering revealed the accumulation trend of the 111 flavonoids in MS-SB-TB comparison. Figure S6: RT-qPCR results for the key genes in phenylpropane pathway. Figure S7: WGCNA of transcriptome data. (A) Cluster dendrogram (B) Module-samples relationships. Figure S8: Co-expression networks related to flavonoids. Red nodes represent the structural genes, green diamonds represent the annotated transcription factors. Figure S9: Ion fragment information of standards and products involved in enzyme assay. Table S1: Information of all detected secondary metabolites in this study (including basic compound informations, classifications, CAS numbers and relative contents in all tissues) In column ‘Compounds’, ‘*’ represent isomers that couldn’t be distinguished. Table S2: List of the metabolites in the 9 clusters of Figure 2F. Table S3: List of the accessions of the genes involved in Figure 3C. Table S4: List of the accessions and annotations of the hub genes and co-expressed transcription factors in Figure S8. Table S5: List of the accessions and annotations of the hub genes and co-expressed transcription factors in Figure 5A.
