# Supplementary material for: Metabolome and Transcriptome Analysis Revealed the Basis of the Difference in Antioxidant Capacity in Different Tissues of Citrus reticulata ‘Ponkan’
Source: Antioxidants (Basel). 2024 Feb 18;13(2):243. doi: 10.3390/antiox13020243 (PMC10886001; doi:10.3390/antiox13020243)
Supplement: Supplementary file 1 [file antioxidants-13-00243-s001.zip › Supplementary Figures-proof.pdf]

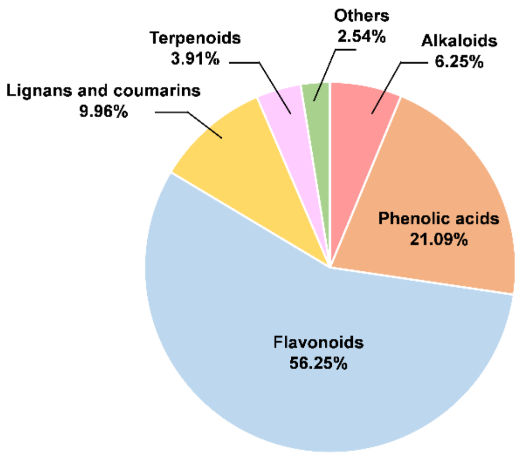

Figure S1. Classification and percentage of the identified secondary metabolites from 12 Ponkan tissues.

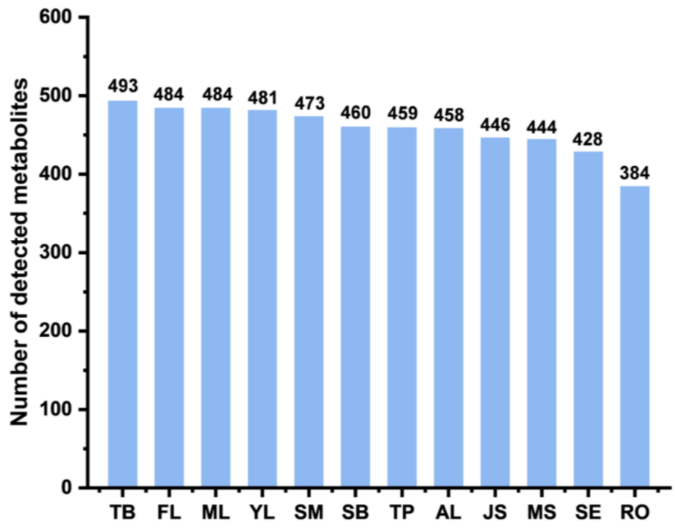

Figure S2. Comparison of the number of secondary metabolites detected in 12 Ponkan tissues.

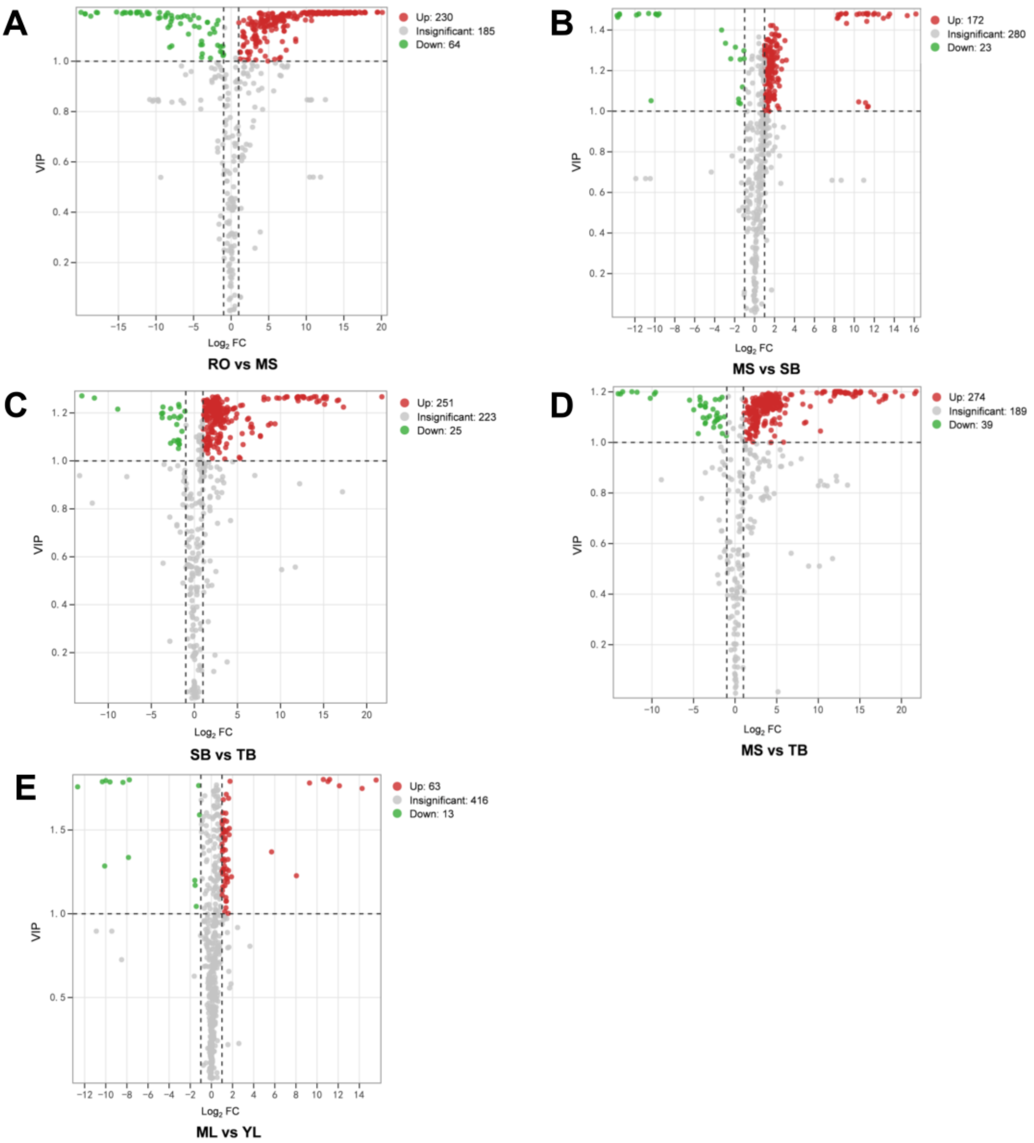

Figure S3. Volcano pots of the DAMs from the comparisons of RO vs MS(A), MS vs SB (B), SB vs TB (C), MS vs TB (D) and ML vs YL (E).

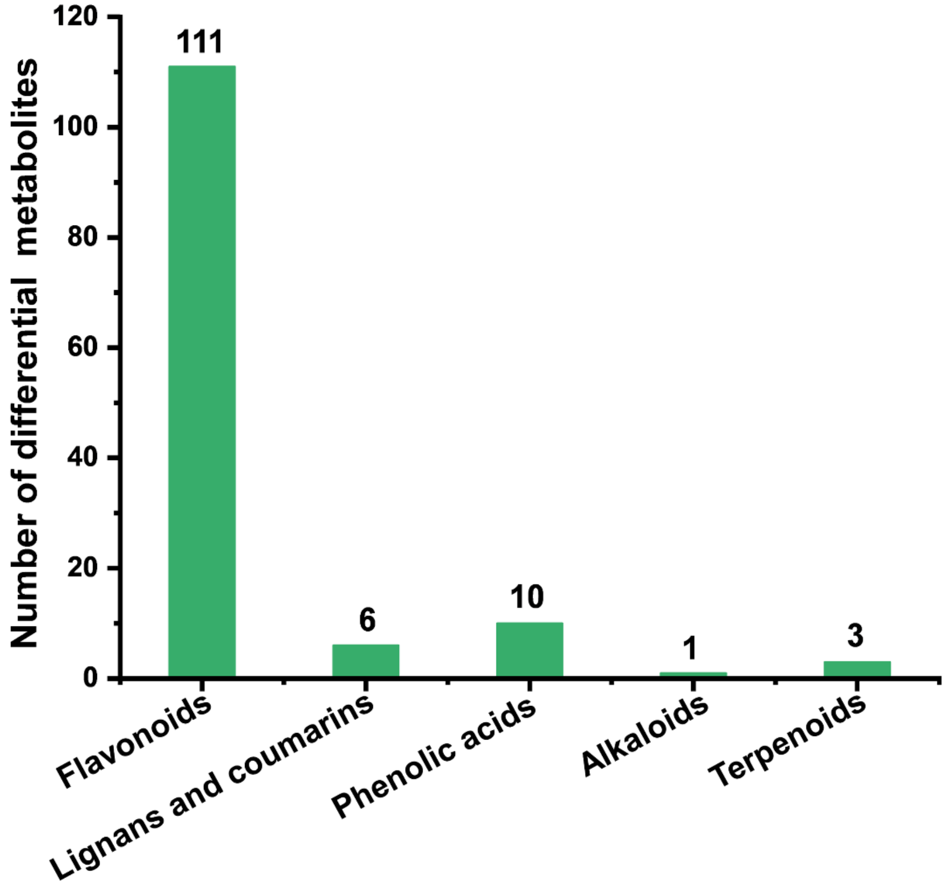

Figure S4. The classification of 131 common metabolites that obtained by pairwise comparison of MS, SB and TB.

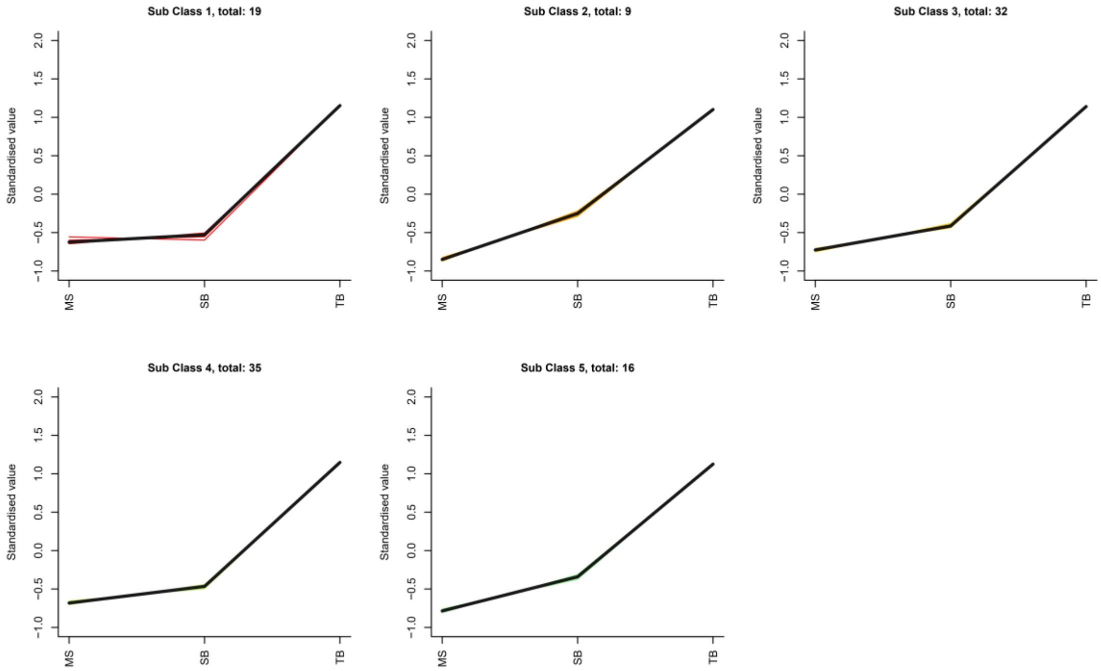

Figure S5. K-means clustering revealed the accumulation trend of the 111 flavonoids

in MS-SB-TB comparison.

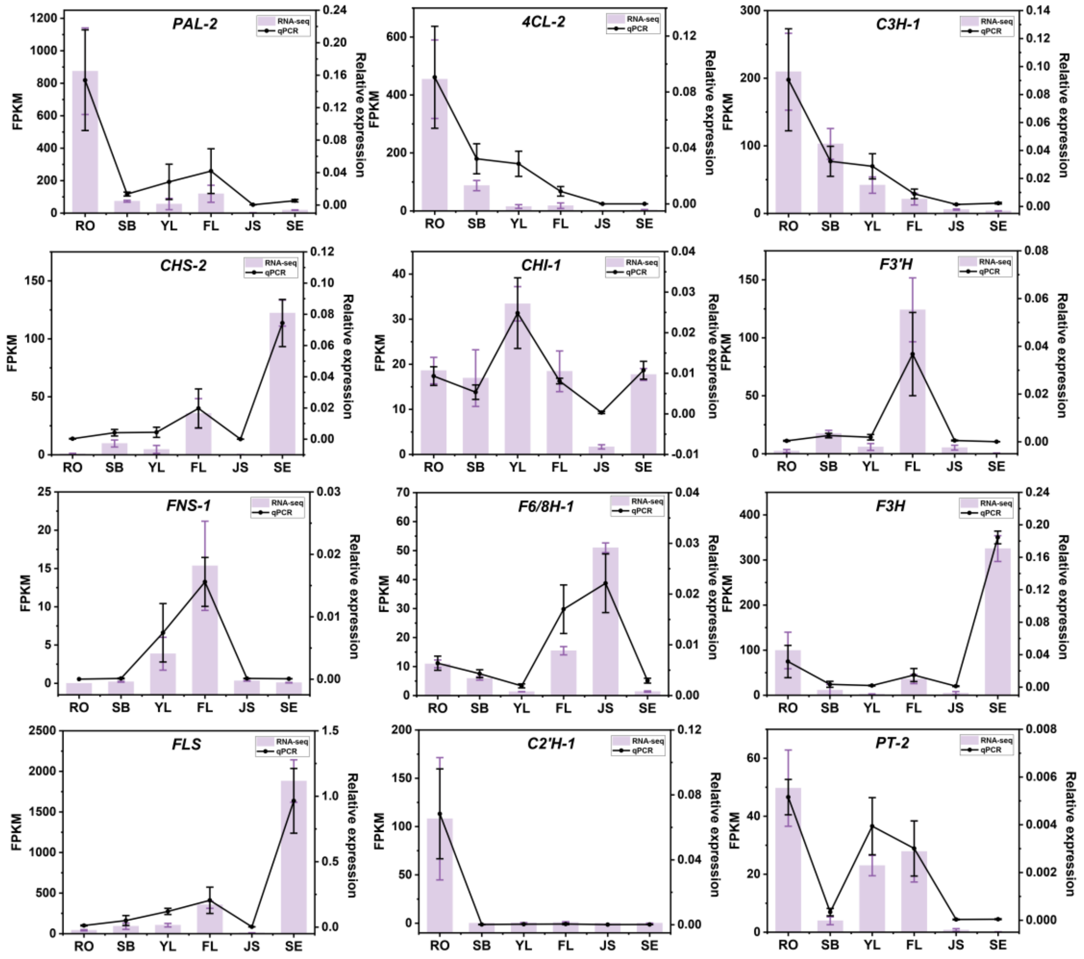

Figure S6. RT-qPCR results for the key genes in phenylpropane pathway.

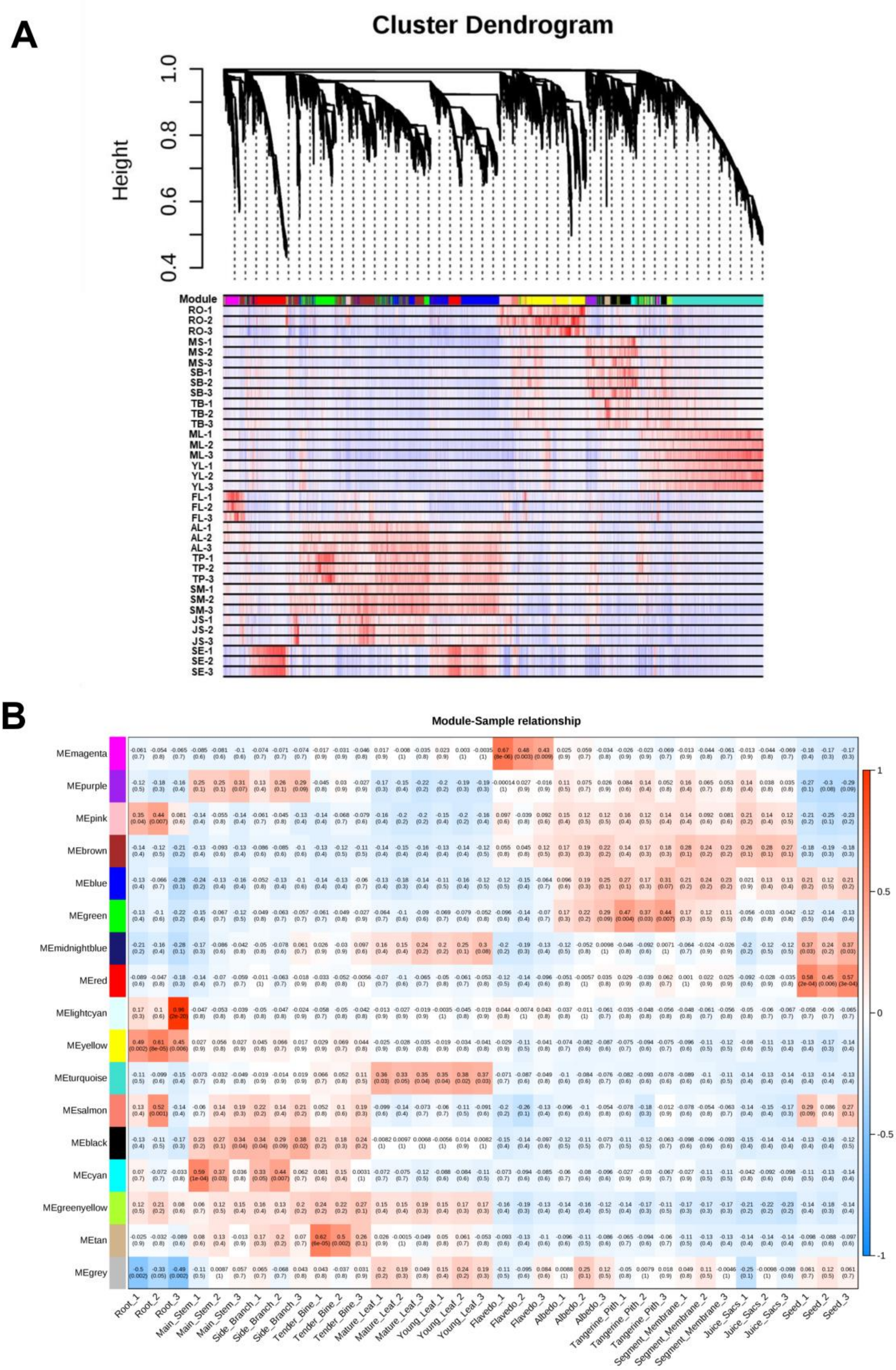

Figure S7. WGCNA of transcriptome data. (A) Cluster dendrogram (B) Module-samples relationships.

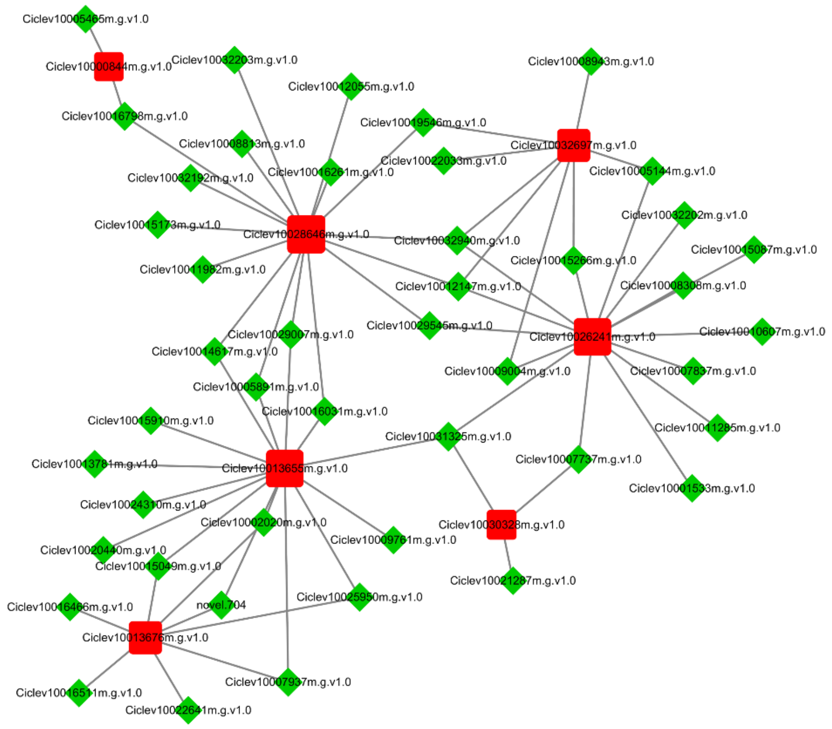

Figure S8. Co-expression networks related to flavonoids. Red nodes represent the structural genes, green diamonds represent the annotated transcription factors.

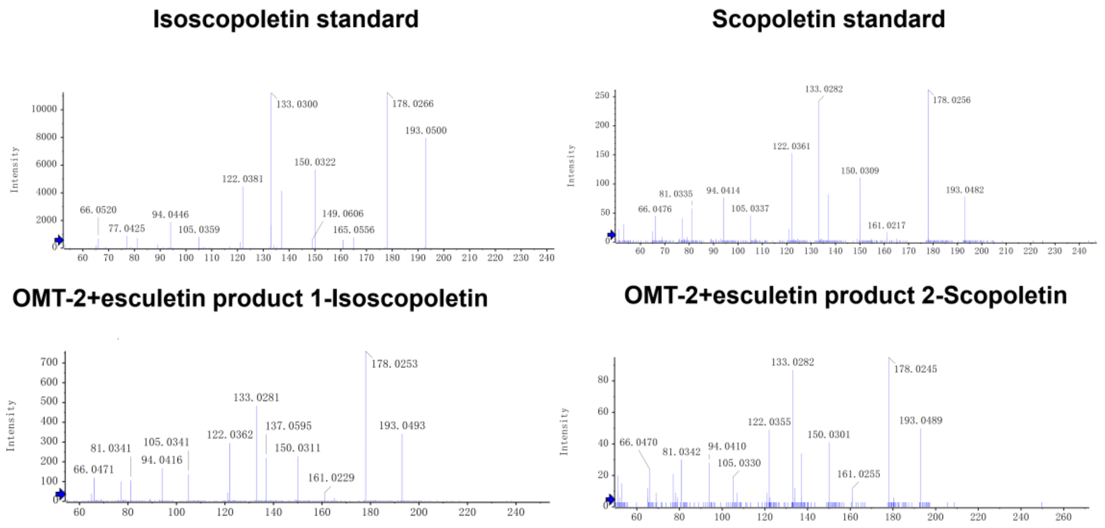

Figure S9. Ion fragment information of standards and products involved in enzyme assay.
